# Incidence of invasive breast cancer in women treated with testosterone implants: a prospective 10-year cohort study

**DOI:** 10.1186/s12885-019-6457-8

**Published:** 2019-12-30

**Authors:** Rebecca L. Glaser, Anne E. York, Constantine Dimitrakakis

**Affiliations:** 1 Millennium Wellness Center, 228 E. Spring Valley Road, Dayton, OH 45458 USA; 2 Department of Surgery, Wright State University Boonshoft School of Medicine, 3460 Colonel Glenn Highway, Dayton, OH 45435 USA; 3 York Data Analysis, 6018 Sycamore Ave NW, Seattle, WA 98107 USA; 4 1st Department of Ob-Gyn, Athens University Medical School, 80 Vas Sophias Street, 11528 Athens, Greece; 5 National Institutes of Health, NICHD, Bldg. 10, 10 Center Drive, Bethesda, MD 20892-1103 USA

**Keywords:** Breast cancer, Incidence, Women, Testosterone, Implants, Prevention

## Abstract

**Background:**

Testosterone implants have been used for over eighty years to treat symptoms of hormone deficiency in pre and postmenopausal women. Evidence supports that androgens are breast protective. However, there is a lack of data on the long-term effect of testosterone therapy on the incidence of invasive breast cancer (IBC). This study was specifically designed to investigate the incidence of IBC in pre and postmenopausal women (presenting with symptoms of androgen deficiency) treated with subcutaneous testosterone implants or testosterone implants combined with anastrozole.

**Methods:**

The 10-year prospective cohort study was approved in March 2008 at which time recruitment was initiated. Recruitment was closed March 2013. Pre and postmenopausal women receiving at least two pellet insertions were eligible for analysis (*N* = 1267). Breast cancer incidence rates were reported as an unadjusted, un-weighted value of newly diagnosed cases divided by the sum of ‘person-time of observation’ for the at-risk population. Incidence rates on testosterone therapy were compared to age-specific Surveillance Epidemiology and End Results (SEER) incidence rates and historical controls. Bootstrap sampling distributions were constructed to verify comparisons and tests of significance that existed between our results and SEER data.

**Results:**

As of March 2018, a total of 11 (versus 18 expected) cases of IBC were diagnosed in patients within 240-days following their last testosterone insertion equating to an incidence rate of 165/100000 p-y, which is significantly less than the age-matched SEER expected incidence rate of 271/100000 p-y (*p* < 0.001) and historical controls.

**Conclusion:**

Long term therapy with subcutaneous testosterone, or testosterone combined with anastrozole, did not increase the incidence of IBC. Testosterone should be further investigated for hormone therapy and breast cancer prevention.

## Background

Breast cancer remains the most common cancer in women worldwide and preventive strategies are in their infancy. Although there is cumulative data supporting the protective role of androgens in breast tissue [1–3], the long-term effect of bio-similar testosterone (T) therapy on the incidence of breast cancer has not been previously documented in a prospective study. This is becoming increasingly important as more studies are being published on the benefits of T therapy in pre and post-menopausal women [3–6].

Subcutaneous T implants have been used to treat symptoms of hormone/androgen deficiency in women since 1937 [6, 7]. It has been known for over 70 years that T is anti-proliferative in the breast and inhibits the stimulatory actions of estrogens. Androgens, including subcutaneous T implants, have been successfully used to treat breast cancer [6–12]. However, there has been some concern about T therapy in women, due to some epidemiologic studies reporting an association (often misinterpreted as causation) between endogenous T levels and breast cancer risk. T is not an independent variable, and many studies do not adjust for associated higher estradiol levels. Furthermore, epidemiological studies do not address the ‘Obesity-Insulin-Testosterone’ connection. Obesity and insulin increase inflammation and have direct and indirect causal effects in breast cancer, including increased aromatase activity [13–15]. Insulin stimulates the production of T, which can account for higher T levels ‘associated’ with breast cancer. In addition, androgen assays lack reliability in the relatively low range of androgens in women, which vary from day to day. Many epidemiologic studies do not measure free bioavailable T levels, which depends on sex hormone binding globulin levels and other endocrine, genetic, and metabolic influences. Also, androgen activity within the mammary cells is not reflected in circulating levels of androgens. Finally, correlation does not imply causation: correlation alone cannot be used as evidence for a cause-and-effect relationship, particularly in view of conflicting studies, and the lack of coherence with biological, preclinical, and clinical evidence.

Testosterone (bio-similar, non-methylated) therapy in women has not been shown to increase the risk of breast cancer and may lower the risk from estrogen-progestin therapy [16–18]. Although the Nurse’s Health Study showed an increased risk of breast cancer in ‘current users’ of oral, methyl-testosterone (the majority of whom were on oral estrogen-progestin therapy), other studies have shown no significant increased risk of breast cancer with methyl-testosterone particularly with esterified estrogens alone (no progestins) [18, 19].

There is concern that T aromatizes to estradiol, which has a secondary stimulatory effect via the estrogen receptor (ER). Patients with increased aromatase activity may produce excess estrogen resulting in breast tissue proliferation [13–15]. Anastrozole, combined with T in a pellet implant, has been shown to prevent aromatization and provide adequate levels of T without elevating estradiol or increasing recurrence in breast cancer survivors [20]. Testosterone has also been shown to safely relieve side effects of aromatase inhibitor therapy in breast cancer survivors [21–24].

The ‘Dayton study’ was specifically designed to investigate the long-term incidence of breast cancer in women treated with T for symptoms of hormone deficiency. Early results were reported at year five as an interim analysis [12]. This 10-year analysis reports the incidence of IBC in women treated with subcutaneous T, or testosterone with anastrozole (T + A), combined in the implant, most often without the concurrent use of systemic estrogen or synthetic progestogens.

## Methods

### Study design, setting, and participants

A 10-year prospective cohort study, investigating the incidence of breast cancer in women treated with subcutaneous T implants, was IRB approved in March of 2008 at which time recruitment was initiated. Recruitment was closed 31 March 2013. Methods, including study design, setting, and participants were previously reported in our 5-year interim analysis published in 2013 [12]:

‘Pre and post-menopausal patients participating in the study were either self-referred or referred by their physician to the clinic (RG) at the Millennium Wellness Center in Dayton, Ohio for symptoms of hormone deficiency or imbalance including hot flashes, sweating, sleep disturbance, heart discomfort, depressive mood, irritability, anxiety, pre-menstrual syndrome, fatigue, memory loss, menstrual or migraine headaches, vaginal dryness, sexual problems, urinary symptoms including incontinence, musculoskeletal pain, and bone loss. Female patients with no personal history of breast cancer were asked to participate in this study. Study size was not predetermined. No patient was excluded from participation based on age, family history, prior hormone use, oral contraceptive use, endometrial pathology, breast density, increased breast cancer risk, menopausal status or body mass index (BMI). Breast cancer genetic testing was not part of the protocol. Although no patient seen during the enrollment period had a known BRCA mutation per clinical history: per protocol they would have been excluded from this group and followed separately. Mammography and clinical breast exam were not protocol determined. Screening mammograms were recommended but not required prior to enrollment. In addition, benefits and risks of screening mammography were discussed with patients. Patients who had received T implants prior to the IRB approval date were not excluded from participation and were recruited to the study beginning March 2008. Patients receiving two or more sets of implants were eligible for analysis (N = 1267). An IRB approved written informed consent was obtained on all patients enrolled in the study. Per protocol, the incidence of breast cancer in our study population was to be compared to historical controls as well as age specific (age matched) Surveillance Epidemiology and End Results (SEER) data. Although a control group was not part of the original IRB approved protocol, it was predetermined (per protocol) that patients receiving only one pellet implant, i.e., three months of therapy, would not be eligible for analysis. Such short-term hormone use would not have a significant impact on the long-term incidence of breast cancer. This group of 119 patients accrued 2008-2009 was followed prospectively through 2013 as an age matched ‘pseudo-control’ group.

In 2013, the institution (Atrium Hospital, Middletown, Ohio) was sold and disbanded their IRB due to restructuring. All accrued patients continued to be followed. A second IRB protocol was approved in April 2018 allowing review and publication of collected data (ClinicalTrials.gov NCT03768128).

### Therapy: subcutaneous testosterone (T) and testosterone combined with anastrozole implants (T + A)

Subcutaneous implants are composed of non-micronized USP testosterone (T) and stearic acid in a geometric ratio of 20:1, or non-micronized USP T, stearic acid and USP anastrozole in a geometric ratio of 15:1:1. Implants are placed in sealed glass vials and autoclaved for sterility and ‘heat fusion’. The sterile implants are inserted into the subcutaneous tissue of the upper gluteal area or lower abdomen through a 5 mm incision using local anesthesia and a disposable trocar kit.

We have previously shown that subcutaneous T therapy alone is able to treat menopausal symptoms in the majority of patients [4]. This is not surprising if one understands basic physiology. T is the major substrate for estradiol in both men and postmenopausal women. The continuous release of T from the implant provides continuous bioavailable T at the cellular level where T has a direct effect via the androgen receptor. In addition, peripheral aromatization of T in fatty tissue and, more importantly, local aromatization at the cellular level, provide adequate and continuous amounts of estradiol at the estrogen receptor. Testosterone implant dosing is weight based with an average starting dose in our study patients of 2–2.5 mg/kg. Patients were evaluated at each visit and T dose was adjusted based on clinical response and side effects. The average interval for T pellet insertion in our study population was 13.8 + 3.8 weeks. Serum total T levels were measured using liquid chromatography tandem mass spectrometry (LC/MS-MS) or by electrochemiluminescence immunoassay (ECLIA) standardized via isotope dilution-gas chromatography mass spectrometry. The methodology used depended on the laboratory, which was determined by insurance coverage. Mean serum T level measured 4-weeks post implantation was 299 + 107 ng/dl (range 101–633, CV 35.9%). Mean serum T level when symptoms returned (trough) was 171 + 73 ng/dl (range 22–461, CV 42.6%), well above endogenous ranges [4, 5]. Doses of T have increased over the past 10 years. More recent data on serum T levels measured one week following implantation demonstrated a mean serum T of 490 + 210 ng/dl (CV 42.8%) on an average dose of 198.7 + 55.8 mg of T (*n* = 398). Despite pharmacologic (therapeutic) serum levels, there have been no adverse events attributed to T therapy other than expected androgenic side effects, which are dose dependent and reversible [4, 5]. As previously reported, 85% of patients reported a mild to moderate increase in facial hair, 6% reported a severe increase in facial hair, 11% reported an increase in acne, 50% reported improvement in skin moisture, tone/texture, and fewer wrinkles, and 1% reported perceived voice changes (voice cracking, raspy voice, or deeper voice) [5].

The clinic began using anastrozole, an aromatase inhibitor, combined in the pellet implant in 2008, initially to treat symptoms of hormone deficiency in estrogen receptor positive breast cancer survivors. The amount of anastrozole in each pellet implant is 4 mg combined with 60 mg of T, providing continuous and simultaneous release of both the T and anastrozole. A dose of 4 to 8 mg of anastrozole (1 or 2 T + A implants) has been shown to prevent elevation of estradiol in breast cancer survivors treated with subcutaneous T [20, 25]. Subsequently, beginning in 2010, women who presented with signs or symptoms of excess estrogen (e.g., breast pain, fluid retention, weight gain, anxiety, irritability), obesity, or increased risk for breast cancer, were treated with anastrozole in combination with T. It was also found that pre-menopausal patients with symptoms of excess estrogen including migraine headaches, dysfunctional uterine bleeding, endometriosis, uterine fibroids, breast pain, or severe premenstrual syndrome also benefited from the ‘low dose’ anastrozole (compared to 1 mg/day oral) delivered subcutaneously with T. T and T + A dosing is based on clinical history and symptoms, response to therapy, weight (BMI), amount of fatty tissue, and laboratory evaluation of hormone levels. There have been no adverse drug events related to subcutaneous anastrozole therapy. The amount of anastrozole released over 100 days is approximately 0.04–0.08 mg per day.

### Data analytics, patient follow up

As previously reported in our 5-year interim analysis [12]:

‘A custom web-based application using Microsoft Active Server Pages with a MySQL database backend system was developed to prospectively follow and track patients. Date and dose of the first T implant insertion and each subsequent insertion along with patient identifiers were entered. The computer program continuously tracks the number of person-days for patient and calculates a running sum (cumulative total) across the group. The system was programmed to identify women who had not returned for therapy within a pre-set time frame of 240 days, 2.5 times the average interval of insertion/duration of clinical efficacy of 96 days. Weekly ‘follow-up’ phone calls were made by designated research personnel. Any participant not seen for 240 days was contacted and breast cancer status was documented. All patients no longer receiving therapy, agreed to contact the office in the future for any subsequent diagnosis of breast cancer.’

Approaching study years 5, 7, 9, and 10, additional phone calls were made to patients no longer on T therapy to document breast cancer status.

All abnormal mammograms were followed until biopsy results were available or subsequent imaging demonstrated a Breast Imaging Reporting and Data System assessment of category 1 (negative) or category 2 (benign, non-cancerous). Any self-reported palpable masses were evaluated by clinical breast exam and office ultrasound (RG) followed by radiographic evaluation and biopsy if indicated. All breast cancers were verified by obtaining pathology reports from core biopsies and definitive surgical procedures.

### Statistical methods

The incidence rates of invasive breast cancer for the Dayton study are reported as an unadjusted, un-weighted value of newly diagnosed cases divided by the sum of person-time of observation of the ‘at risk’ population. Person-days of observation were calculated from the date of first T pellet insertion for each participant up to the date of cancer registration, the date of death, 240 days after the last pellet insert, or the set date of 31 March 2018, whichever came first. A cumulative total of ‘person-days’ was calculated for the predetermined 240-day horizon. Person-years (p-y) were calculated by dividing person-days by 365.25. The incidence of breast cancer was calculated per 100,000 p-y so that our results could be compared to age matched SEER breast cancer incidence rates and historical studies*.*

Unlike pills and topical therapy, subcutaneous implants are long acting (sustained release) and the incidence of breast cancer was reported for predetermined time frame of 240-days post implantation or 2.5 times the average length of clinical efficacy of 96 days [3].

The observed breast cancer incidence rates for the Dayton study patients were compared to the expected (adjusted) SEER breast cancer incidence rates calculated from the age composition of Dayton study patients and the published SEER age-grouped breast cancer incidence rates for two time periods, 2007–2011 and 2011–2015 [Additional file1, Table 1s]. This approach allowed for the possibilities of changing cancer rates over the course of the study and the change in the age composition of our study patients. The ‘expected incidence’ is a weighted sum of the SEER incidence rates with the weights corresponding to the proportion of the Dayton study patients’ person-years (p-y) in each of the SEER age groups.

**Table 1:** Patient demographics at first T pellet insertion [12]

	*N* = 1267
Postmenopausal	76.8%
Surgical %	66.2
Natural %	43.8
Pre/perimenopausal	23.2%
Age, mean (SD)	52.1 + 8.6 y
Family history BCA (1st, 2nd)	29%
Age menarche, mean (SD)	12.8 + 1.6 y
Age first birth, mean (SD)	24.8 + 5.2 y
Nulliparous	14.9%
Weight kg, mean (SD)	71.03 + 15.5 kg
BMI, mean (SD)	26.3 + 5.5 kg/m^2^

Classical estimates of the incidence rates and the expected incidence rates, based on the assumption that breast cancer numbers followed a Poisson distribution, were derived. In order to verify the assumptions, bootstrapped estimates of these quantities were calculated. Both bootstrapping and the classical methods were used to assess the significance of these results [26]. For the bootstrapped experiments, 10,000 pseudo-replicates were drawn, and breast cancer incidence rate calculations were repeated. From this ensemble of “replicates” the distributions of incidence were estimated along with expected SEER incidence rates. These were compared to the classical estimates. In addition, asymptotic estimates of the differences between our rates and the expected SEER rates, and the ratios of the SEER rates to our estimated rates were derived. Estimated confidence intervals for Dayton and expected SEER rates and numbers of cancers, and significance tests of difference were performed. All calculations were performed in R [27]; the bootstrapped confidence intervals and tests of statistical significance followed from the R package boot version 1.3–9 [Additional file 1] [27].

## Results

### Patient demographics, accrual

As of March 2013, 1267 patients had been accrued to the study and were eligible for analysis having received more than one pellet implant. Patient demographics at initial T insertion are listed in Table 1. Characteristics of the study population were similar to women of a comparable age in the United States. Patients were not at an increased or decreased risk for breast cancer based on family history, hormonal, or reproductive factors.

The majority of patients (62%) were accrued to the study within the first year. Over 85% of patients were accrued by study year 2, 90% by year 3, and 96% by year 4 [12]. The mean length of T therapy through March 2018 was 5.3 + 3.5 y (range 0.7–12.2 y), which included women who received their first T implant prior to study accrual.

407 out of 1267 patients continued to receive therapy March 2017 through March 2018. Current patient demographics are listed in Table 2. The oldest patient was 91.7 years old. The youngest patient was 37.2 years old and has been treated continuously since October 2007 for intractable migraine headaches.

**Table 2:** Patient demographics, current users of T therapy

	*N* = 407
Menopausal status, N (percent)	
Premenopausal	43 (10.6%)
Postmenopausal	364 (89.4%)
Age, mean (SD), (range)	
1st insert	51.7 + 8.1 y (27.4–80.0)
Current	61.1 + 8.4 y (37.9–91.7)
Weight, mean (SD)	
1st insert	69.6 + 15.1 kg
Current	69.8 + 13.6 kg
Length of T therapy, mean (SD)	9.34 + 1.71 y
Current T dose, mean (SD)	192 + 50 mg
Aromatase inhibitor use, N (percent)	
Total	86 (21.1%)
Premenopausal	27 (62.8%)
Postmenopausal	59 (16.2%)

The percent of female patients treated with the combination T + A implant increased from approximately 11% in 2010, to 30% January through July 2011, and to a maximum of 62% December 2012 through March 2013. As women have aged and transitioned into menopause, thus producing less estrogen, fewer women require the addition of the aromatase inhibitor. Currently 21.1% of study patients are treated with anastrozole combined in the implant: 16.2% of postmenopausal patients and 62.8% of premenopausal patients.

### Breast cancer incidence

As of March 2018, there have been 11 cases of IBC diagnosed in women within 240 days following their most recent T implant insertion in 6667 p-y of therapy, which translates to an incidence of 165/100000 p-y. The incidence of IBC in women treated with T therapy was significantly less than our previously reported ‘control’ group incidence of 390/100000 p-y, *P* < 0.001 [12]. No patient was diagnosed with breast cancer within the first 240 days following (their) initial T pellet insertion.

### Comparison to SEER data and historical controls

Significantly fewer cases of IBC were diagnosed in our study group compared to the age-matched SEER expected number of IBC cases. At the 240-day designated time period, 11 cases of IBC were diagnosed in our patient population compared to 18 cases expected based on age-matched SEER data [28].

The age-matched SEER incidence rate for IBC was 271/100000 p-y. The calculations are presented in detail in Additional file 1. Asymptotic estimates of Dayton and expected SEER incidence rates (N/100000 P-Y) are shown in Table 3. There was a 39% reduction in the incidence of breast cancer in patients on T therapy compared to the expected age-matched SEER incidence rate (*p* < 0.001). Estimated confidence intervals for Dayton and expected SEER rates and numbers of cancers, and significance tests of difference for whole sample results are presented in Additional file 1, Tables 4 and 5.

**Table 3:** Asymptotic estimate of Dayton and expected age-matched SEER incidence rate (per 100,000 p-y), standard deviations (SD), the ratio of the Dayton incidence rate to the SEER and it’s SD. See Additional file 1 for the details of the method of calculation

Horizon	Events	P-Y	Dayton incidence	Dayton SD	Seer incidence	Seer SD	Ratio	Ratio SD
240 d	11	6666.6	165.0	49.8	270.5	1.83	0.61	0.18

**Table 4:** Incidence rates of IBC, comparison to published studies

	Cases per 100,000 p-y	Years Observed
Dayton Study		
T, T + AI	165	10
WHI RCT ^29,30^		
Placebo	330	10.7
E alone	260	10.7
E + P	380	5.2
MWS ^31^		
Never users	312	14
E alone, E + P	501	14
Adelaide ^14^		
T + E, T + E + P	238	5.9
T + E + P	293	5.9

**Table 5:** Patient data and tumor characteristics, twelve patients treated with T or T + A implants diagnosed with invasive breast cancer March 2008–March 2018 within 240 days of receiving therapy

Patient	Age at 1st TT Y	Age at IBC dx. Y	BMI 1st TT	Meno Status 1st TT	Prior E use	Detection	N days Last insert prior to dx	IBC (Stage) Type	Receptor status	Continued TT post dx
1	46.2	49.3	19.2	TAH FSH 4.6	Y	Mammo	206 d	T1b, N0 (1) Gd 2 IDC	ER+, PR+ Her 2 -	
2	55.0	59.2	33.3	Post	Y	Palpable	123 d	T3, N2 (3) Gd 3 IDC	ER-, PR-Her 2 -	
3	50.0	52.9	19.2	Pre	OCP Current	Mammo	34 d	T1c, N0 (1) Gd 1 IDC	ER+, PR+ Her 2 -	T + AI x 5y T alone
4	67.6	70.2	24.7	TAH BSO	Y	Mammo	151 d	T1b, N0 (1) Gd 1 IDC	ER+, PR+ Her 2 –	
5	44.9	48.5	21.5	TAH	N	Mammo	48 d^a^	T1c, N0 (1) Gd 1, IDC	ER+, PR- Her 2 -	
6	48.9	55.5	24.4	TAH	Y	Mammo	146 d	T1b, N1a (2) Gd 2 ILC	ER+, PR- Her 2 -	
7	56.2	60.6	28.8	TAH BSO	Y	Mammo	51 d	T1 N0 (1) Gd 2 IDC	ER+, PR+ Her 2 -	
8	50.0	55.2	28.7	Post	N	Mammo	54 d	T1a N0 (1) 1.2 mm IDC	ER-, PR+ Her 2 -	T alone
9	58.0	61.5	39.3	TAH BSO	N	Palpable	50 d	T2 N1 (2) Gd 3 IDC	ER+ PR+ Her 2 -	T + AI Note: tested BRCA 2 pos.
10	39.7	43.4	23.2	Pre	N	Palpable	15 d	T1c N0 (1) Gd 2 IDC	ER+ PR+ Her 2-	
11	44.0	51.0	23.5	Pre	N	Mammo	92 d	Clinical T2 N0 (2A) Gd 3 IDC	ER+ PR+ Her 2 +	T + AI

^a^Patient was diagnosed 48 d after a single pellet insertion following a 23-month lapse in therapy

Abbr: TT testosterone therapy, IBC (invasive breast cancer), Dx. (diagnosis), BMI (Body mass index), E2 (estradiol), OCP (Oral Contraceptive Pill), IDC (Infiltrating ductal carcinoma), ILC (infiltrating lobular carcinoma), T (tumor size), a (< 0.5 cm), b (> 0.5, < 1 cm), c (> 1, < 2 cm), T2 (20mm–50mm), T3 (> 50 mm), N (node status); N0 (no nodes positive), N1a (Single node < 5 mm), N2 (4–9 nodes positive), Gd (tumor grade: 1 low grade, 2 intermediate grade, 3 high grade), ER (estrogen receptor), PR (progesterone receptor), HER2 (human epidermal growth factor 2), T (testosterone implant), T + AI (testosterone combined with an aromatase inhibitor implant), Mammo (mammography)

In addition, our 10-year incidence rates are lower compared to previous studies using conventional hormonal (estrogen/progestin) regimens including the Adelaide Study, which previously reported a reduced incidence of breast cancer with subcutaneous T used in combination with conventional hormone therapy, Table 4 [17, 29–31].

Abbrev: *T* testosterone, *E* estrogen, *P* progestin, *WHI RCT* Women’s Health Initiative Randomized Control Trial, *MWS* Million Woman Study.

### Bootstrap results

**Fig. 1:**
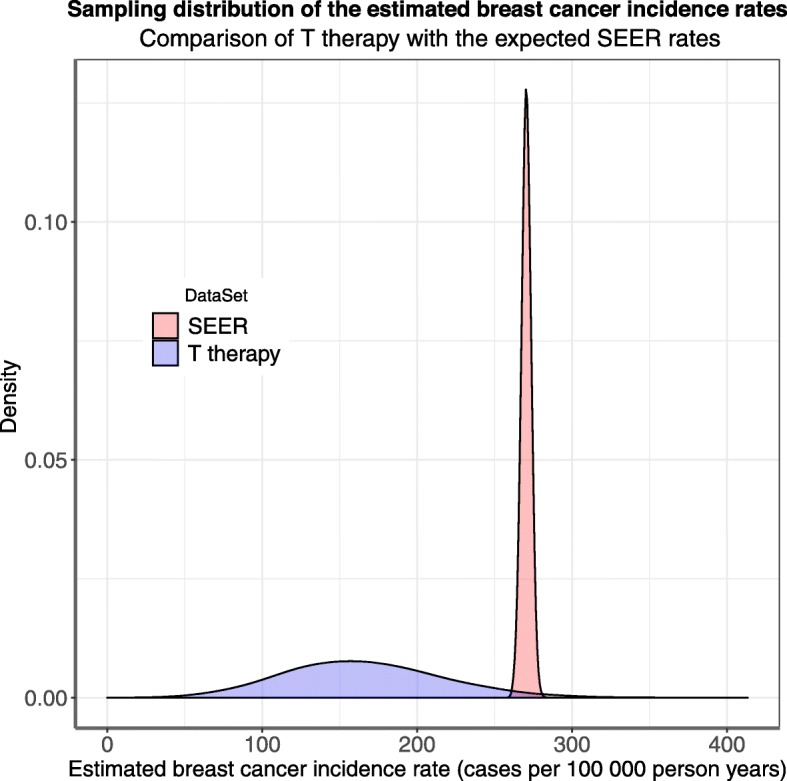
Bootstrap results confirm a significant reduction in IBC on T therapy

Bootstrap results confirm a marked reduction in the incidence/distribution of invasive breast cancer at 10-years in T and T + A users (Fig. 1). Estimated confidence intervals for Dayton and expected SEER incidence rates and numbers of cancers, and significance tests of difference for bootstrap results are presented in Additional file 1, Tables 4 and 5.

### Breast cancer characteristics

Patient data and tumor characteristics of the 11 women diagnosed with invasive breast cancer are presented in Table 4. Mean age at first insert was 50.97 + 7.44 y. The mean age at diagnosis was 55.22 + 7.42 y. The mean length of therapy prior to diagnosis was 4.25 y (range 2.60–6.96 y). Eight of 11 cancers were diagnosed on screening mammography. The three patients that were diagnosed with palpable tumors did have screening mammograms within 1–2 years of diagnosis. Nine of 11 tumors were estrogen receptor (ER) positive. Seven of 11 were stage 1. Of interest, patient 11 was diagnosed with an ER positive tumor while on T / T + A therapy. She received implants containing T (180 mg) plus Letrozole (12 mg) before starting neoadjuvant therapy and there was a 43% reduction in tumor volume within 41 days after implantation *prior* to receiving systemic chemotherapy. The patient continued T + Letrozole throughout chemotherapy and had a complete pathologic response. T also attenuated many side effects of chemotherapy [9]. Three other patients diagnosed with invasive breast cancer have also continued on T implant therapy. As of March 2018, all patients are alive and well with no evidence of disease.

### Ductal carcinoma in situ

From March 2008 through March 2018 three patients enrolled in the study were diagnosed with ductal carcinoma in situ (DCIS) within 240 days of their last pellet insertion. Mean age at the first T pellet insert was 57.74 + 3.73 years. Mean age at diagnosis of DCIS was 64.44 + 4.07 years. The incidence of DCIS in our study populations was 45/100000 p-y compared to the SEER expected incidence rate for DCIS of 84/100000 p-y for women age 60–64 [28].

## Discussion

Our 10-year analysis of the Dayton study demonstrated a 39% lower incidence of (invasive) breast cancer in T users compared to the ‘age-matched’ SEER expected incidence. This was not surprising. Although a detailed discussion of the favorable effect of T in the breast is beyond the scope of this paper, it is known that T’s direct effect at the androgen receptor (AR) is antiproliferative, proapoptotic, and inhibits ER α and breast cancer growth [1, 3]. Clinical studies in primates and humans support the inhibitory effect of T in the breast [1–3]. We have demonstrated remarkable responses (clinical exam, mammography, ultrasound) of hormone receptor positive tumors to T + aromatase inhibitor implant therapy in the neoadjuvant setting, further confirming the direct beneficial effect of T at the AR [8, 9]. T also improves glycemic control and attenuates the inflammatory process, both of which could have a beneficial effect on the incidence of breast cancer [14, 15, 32, 33].

Short-term studies on transdermal T therapy have not shown an increase in the incidence of breast cancer nor did they demonstrate a reduced incidence [3, 16]. Subcutaneous implants provide continuous delivery of therapeutic levels of T and results from T implants may not be applicable to other methods of delivery or lower doses of therapy [4, 5, 24]. Also, patients differ in their ability to aromatize T to estradiol [14, 15]. Caution should be used in treating patients with clinical evidence of increased aromatase activity and consideration should be given to the addition of aromatase inhibitor therapy when indicated.

This prospective study was specifically designed to investigate the incidence of breast cancer in a relatively large sample size. Demographic characteristics of our sample are those of a normal population and are not women at low or high risk for breast cancer. All study patients were followed by a breast surgeon (RG), and the vast majority of women over 40 years of age underwent screening mammography, which could have increased the incidence of detected IBC’s. Our study is unique in that most (over 95%) of women were treated with T implants alone without systemic estrogen or progestogens. However, some women did receive estrogen therapy prior to T alone, which could have affected the incidence of IBC. A major strength of our study is that patients were evaluated at each office visit when they received the implants, and there are no missing data on incidence of breast cancer in these patients. Exact doses and intervals of insertion were documented in patient charts allowing for known levels of exposure to the drug therapies, as well as any medical changes, diagnoses, or adverse events. There was no potential for unknown non-compliance to therapy in contrast to other studies using oral or topical formulations where ‘use of hormone therapy’ is often self-reported from memory or from data on ‘filled’ prescriptions.

There is some controversy regarding serum levels of T on subcutaneous implant therapy. We have previously published data on ‘efficacy and safety’, ‘inter-subject variability in serum levels’, as well as detailed rationale supporting the doses of T used (for over ten years) in our patient population [4, 5]. Although (peak) serum T levels, measured one-week post implantation, were in the lower range for endogenous production in men, this has proven inconsequential. Pharmacologic levels in serum do not equate to supra-physiologic levels at the end organ androgen receptor (AR). On the contrary, we have shown that pharmacologic T levels in serum equate to physiologic (therapeutic) levels at the AR as evidenced by clinical efficacy and the lack of adverse events, other than androgenic cosmetic side effects, which are reversible with lowering the dose [4, 5]. Specifically, our previous published prospective study showed no voice changes on these doses and levels on therapy [34]. In addition, we have shown scalp hair regrowth on T therapy [35]. The clinical necessity of higher (therapeutic effective) serum levels of T can be explained by the significant age-related decline of the adrenal pro-androgens in addition to T, contributing to a marked reduction of bioavailable T at the cellular level. The amount of T released from the implant (and subsequently measured in serum), is replacing T as well as the significant local contribution of DHEA and androstenedione to bioavailable T at the AR [5]. T’s effect is dose dependent, and there is no evidence (i.e., drug-concentration in blood studies), or documented safety concerns, supporting the ‘opinion’ that serum T levels on therapy should remain within endogenous, or ‘physiologic’ ranges. In point of fact, concentration-response studies on subcutaneous T implants demonstrate the opposite [4, 5, 36]. Most notably, in our patient population, mean serum T level when symptoms returned (trough) was 171 + 73 ng/dl, well above endogenous ranges, with significant inter-individual variation (CV 42.6%). In regard to safety, long-term studies on transgender men have shown that even higher (male) doses of T do not increase the risk of cardiovascular events, stroke, or cancer; T therapy also increases insulin sensitivity, as opposed to estrogen therapy in transgender women [32, 37, 38]. No other hormone medication (e.g., insulin, estrogen) is dosed or micromanaged based solely on levels of the active pharmacologic ingredient in serum, but rather on clinical response/beneficial effects versus adverse side effects. Studies showing ‘lack of efficacy’ may be due to inadequate amounts of T at the AR. Likewise, the breast protective effect demonstrated in our study may not be seen with lower doses of T.

A critique of our study was the use of ECLIA (assay) for measuring T levels in some women. ECLIA is a direct immunoassay without any preceding purification steps, which could result in potential interference from T metabolites.

A limitation of the Dayton study was a lack of a matched control group from the onset. However, this study was never designed as a randomized drug trial. Our results represent ‘real world’ data from a clinical practice where women suffering from symptoms of androgen deficiency received therapy. Another critique of our practice was the use of low dose, subcutaneous anastrozole in some pre/perimenopausal and postmenopausal patients. Our interventions were based on the previously successful use of aromatase inhibitors to treat breast and gynecologic diseases where pathological tissues overexpress aromatase and increase local production of estrogens [13–15]. We have found that low dose anastrozole (0.04–0.08 mg released per day) combined with T delivered subcutaneously effectively treats these conditions without adverse effects or alteration of menstrual cycles [4, 5, 20]. Individual patients were evaluated at each office visit and many women have been treated (alternately) with T or T + A depending on clinical status and symptoms, making it impossible to evaluate the two regimens separately, which remains a major limitation in the interpretation of our results. The potential individual or separate effect of anastrozole on the incidence of IBC remains unknown. However, our earlier results support a protective effect of T, as much of the data was accrued prior to the routine use of anastrozole [12].

Our 10-year results are consistent with preclinical and clinical evidence indicating that androgens have a protective role in the breast and refute the causal interpretation of epidemiologic studies reporting an association of endogenous T levels with IBC. Although subcutaneous T implants have been used (safely) to treat symptoms and diseases, including breast cancer, in women since 1937, there is no FDA approved bio-similar T formulation available for women in the United States. This ‘real world’ long-term data on subcutaneous implants further supports the safety of T therapy in women.

## Conclusion

Our 10-year results demonstrated a 39% reduction in the incidence of IBC in our population compared to the age-matched SEER expected incidence. Long-term subcutaneous T and/or T + A therapy, used to treat symptoms of androgen deficiency in pre and postmenopausal women, did not increase the incidence of invasive breast cancer. Although not novel, testosterone implants should be further investigated for hormone therapy as well as breast cancer prevention. Additional studies, including long-term controlled trials in women treated with testosterone implants alone, and testosterone with anastrozole in a uniform administration, would be optimal to further delineate the effect of T (alone), or T combined with an aromatase inhibitor (when indicated), on the incidence of IBC.

## Supplementary information

**Additional file 1.** Statistical Methods and Results

## Data Availability

All statistical data and analysis are included in Additional file 1: Statistical Methods and Results. Confidential access to primary data including de-identified spreadsheets will be granted upon reasonable request.

## Abbreviations

DCIS: Ductal carcinoma in situ
ER: Estrogen receptor
IBC: Infiltrating Breast Cancer
p-y: Person-years
SEER: Surveillance Epidemiology and End Results
T + A: Testosterone and anastrozole
T: Testosterone
