# Supplementary material for: Incidence of invasive breast cancer in women treated with testosterone implants: a prospective 10-year cohort study
Source: BMC Cancer. 2019 Dec 30;19:1271. doi: 10.1186/s12885-019-6457-8 (PMC6937705; doi:10.1186/s12885-019-6457-8)

Incidence of breast cancer in women treated with testosterone implants: a prospective 10-year cohort study
Supplement 1: Statistical Methods

R. L. Glaser and A. E. York and C. Dimitrakakis

August 5, 2019

Abstract

Summary of statistical methods for describing the uncertainty about the estimates of breast cancer incidence rate and the comparison to the SEER bc incidence rate.

1 Introduction

The observed breast cancer incidence rate for the Dayton study patients was compared to the expected incidence rates calculated from the age composition of our study patients and the published SEER age-grouped breast cancer incidence rates for two time periods, 2005-2011 and 2011-2016 ([1, 2], in Table 1). Although official accrual to the 10- year study began in 2008 and lasted until March of 2013, 407/1388 patients received their first pellet implants prior to this date (2005, n=2; 2006 n= 144; 2007 n=260). No breast cancers were diagnosed between November 2005 and January 2008. The evaluation lasted until March 2018. We assumed that the 2011-2015 breast cancer rates also applied to data collected in 2017 and 2018. This approach allowed for the possibilities of changing cancer rates over the course of the study and the change in the age composition of our study patients.

In this supplement, using theoretical statistics([3]) , we provide formulas for the expected SEER breast cancer incidence rate and its standard deviation based on the age structure of the Dayton study from the national

reported age-grouped breast cancer statistics in [1, 2]. We then, compute the difference between the Dayton and expected SEER incidence rates and the ratio of the Dayton to the SEER incidence rates, and the standard deviations of these quantities. In addition, we compute bootstrap estimates of the same quantities and compare their distribution to the asymptotic results from classical statistics ([4, 5].

2 Methods

The incidence rates of breast cancer (BC) for the Dayton study are computed as unadjusted, un-weighted value of newly diagnosed cases divided by the sum of person-time of observation of the at risk population. Person-days of observation were calculated from the date of first T pellet insertion for each participant up to the date of cancer registration, the date of death, or the set date of 31 March 2018, whichever came first.

Let y_{ij} be the number of person years of active therapy observed for age group i during time period j and s_{ij}, the corresponding SEER breast cancer incidence rate, and N the number of cancers observed in the Dayton study during the period of "active therapy". As the pellets do not stop working immediately after the last insertion, "active therapy" can be any time period for which the pellets are assumed to be effective. In [6], this was defined as 240 days post pellet insertion. Define μ_{Dayton} as the observed incidence rate for a given period of active therapy and μ_{SEER} as the corresponding SEER incidence rate.

$$\mu_{Dayton} = \frac{100000 \quad N}{\Sigma y_{ij}} \tag{1}$$

The proportion of total person-years observed for age group i during time-period j (p_{ij}) (for a given definition of active therapy) is:

$$p_{i,j} = \frac{y_{i,j}}{\sum y_{i,j}} \tag{2}$$

Then, the expected breast cancer rate based on Table 1 is the weighted average of the SEER incidence rates:

$$\mu_{SEER} = \sum s_{i,j} p_{ij} \tag{3}$$

The standard deviation of the observed incidence rate was calculated assuming the number of cancers follows a Poisson distribution so that, $SD(N) = N^{.5}$ and therefore,

$$SD(\mu_{Dayton}) = \frac{100000 * N^{.5}}{\sum y_{ij}} \tag{4}$$

The variance of the expected incidence rate ($_{SEER}$) was estimated in two ways. The first using weighted sum of the estimates of SEER incidence SDs. (Table 3),

$$Var(\mu_{SEER}) = \sum (SD_{i,j} * p_{i,j})^2 \tag{5}$$

and the second, using the empirical sample estimates

$$Var(\mu_{SEER}) = \sum p_{i,j} \ (s_{i,j} p_{i,j} - \mu_{SEER})^2 \tag{6}$$

We have calculated (Table 2) the expected values and their variances for a range of levels of "active therapy" (90 - 365 d).

For comparing the observed incidence rates from the Dayton study to the published SEER rates, we calculated the mean and variance of the difference between the SEER and Dayton study incidence rates and the ratio of the Dayton study and the SEER rates:

Let D be the difference between the observed breast cancer incidence rate and the expected rate based on SEER assumptions ($\mu_{Dayton} \ - \ \mu_{SEER}$). We assume that the SEER and Dayton incidence rates are statistically independent, then the expectation of their differences is the difference of their expectations and the variance of their difference is the sum of their variances:

$$E(D) = \mu_{Dayton} \ - \ \mu_{SEER} \tag{7}$$

$$Var(D) = Var(\mu_{Dayton}) + Var(\mu_{SEER}) \tag{8}$$

If R is the ratio of the observed breast cancer incidence rate to the expected rate based on SEER distribution, assuming that the SEER and the Dayton rates are independent, then

$$R \approx \frac{\mu_{Dayton}}{\mu_{SEER}} (1 + \frac{Var(\mu_{SEER})}{\mu_{SEER}^2}) \tag{9}$$

$$Var(R) \approx (\frac{\mu_{Dayton}}{\mu_{SEER}})^2(\frac{Var(\mu_{SEER})}{\mu_{SEER}} + \frac{Var(\mu_{Dayton})}{\mu_{Dayton}}) \tag{10}$$

In addition to theoretical estimates of mean and variance, bootstrap simulations were performed to verify our estimates of the sampling variability of the breast cancer incidence rates for our study and to determine if the Dayton incidence rates were significantly different from the SEER rates.

In bootstrapping, a collection of "pseudo replicates" was constructed by sampling from the original data. We drew 10,000 pseudo-replicates by sampling with replacement from our study population. The purposes of the bootstrap are to verify the summary statistics for the classical estimates of the Dayton and expected SEER BC incidence rates and to determine their distributions. Each pseudo-replicate is the same size as total sample size of the study population (1,267); it might include a particular patient multiple times or it might not; it may include 0 patients with breast cancer, or it may include many more. From this ensemble of "replicates", we estimated both the distribution of the Dayton incidence and the expected incidence rate assuming that our population followed the SEER rates. From those distributions, we calculated the summary statistics to compare to the classical estimates; these parameters included the means, standard deviations, the difference between the Dayton rates and the expected SEER rates, and the ratio of the SEER rates to the estimated Dayton rates. In addition, see below, we estimated the power of the various hypothesis tests of interest.

Confidence intervals of the Dayton incidence rates based on the whole sample were computed from the Poisson assumption using the R function for the Poisson exact test based on procedures in [7], Chapter 6. Tests of the null hypothesis that Dayton and expected SEER rates were the same were carried out with the same procedure. Percentile confidence intervals from bootstrapped distributions were computed using the methods in [5] (Chapter 12 and 13) and tests of hypothesis from using the [5] (Chapter 16).

The power of a statistical test is the probability that the test will not reject a true null hypothesis. We did not conduct an a priori power analysis and did not conduct a post hoc power analysis because they are open to valid criticism. We believe that the width and non-overlapping nature of

the confidence intervals of our results is evidence that the sample size of our study was sufficient to compare both the Dayton incidence rates with the expected SEER rates and the number of observed cancers with the expected number if the SEER incidence rates were operative.

3 Results

The summary statistics for the single sample were computed using Equations 1 - 10 (Table 2). The distribution of the Dayton and expected SEER incidence rates were also estimated using bootstrap methods. These were computed by drawing with replacement, a sample of size 1,267 from the Dayton patient population and computing the distribution for the Dayton and expected SEER rates using the same formulas as for the complete sample. Summary statistics for the 10 000 bootstrap simulations are in Table 3. These bootstrap experiments were repeated for each value in the range of active therapies of interest. The theoretical single-sample and bootstrap estimates are compared (Tables 2 and 3) for a range of possible values of active therapy.

Confidence interval and results of hypotheses tests for both the whole sample and bootstrap cancer incidence rates (Table 4) and number of cancers (Table 3) show that the Dayton incidence rates and numbers of cancers were uniformly less than expected rates and numbers, assuming the SEER rates were operative. There is no overlap between the Dayton and SEER confidence intervals (Table 4, Figure 1) for the shorter time-periods ($<$ 240 days) and little overlap for the longer. The bootstrapped results tended to be sharper with somewhat shorter confidence intervals.

Confidence interval and hypotheses tests for both the whole sample and bootstrap cancer incidence rates are in Table 4. In all cases, the Dayton and expected SEER incidence rates are significantly different ($P < 0.04$) in all cases, with stronger results for the shorter time-periods. There is no overlap between the Dayton and SEER confidence intervals Figure 1 for the shorter time-periods ($<$ 240 days) and little overlap for the longer periods. The bootstrapped results tended to be sharper with somewhat shorter confidence intervals.

References

[1] Kohler BA, Sherman RL, Howlader N, Jemal A, Ryerson AB, Henry KA, et al. Annual report to the nation on the status of cancer, 1975-2011, featuring incidence of breast cancer subtypes by race/ethnicity, poverty, and state. JNCI: Journal of the National Cancer Institute. 2015;107(6).

[2] Howlader N, Noone A, Krapcho M, J G, Miller D, Altekruse S, et al.. SEER Cancer Statistics Review, 1975-2011, National Cancer Institute. Bethesda, MD; 2016.

[3] Mood AM, Graybill FA, Boes DC. Introduction to the Theory of Statistics. 3rd ed. McGraw-Hill Series in Probabliity and Statistics. McGraw-Hill; 1974.

[4] Davison AC, Hinkley DV. Bootstrap Methods and Their Applications. Cambridge: Cambridge University Press; 1997. ISBN 0-521-57391-2. Available from: http://statwww.epfl.ch/davison/BMA/.

[5] Efron B, Tibshirani RJ. An Introduction to the Bootstrap. No. 57 in Monographs on Statistics and Applied Probabliity. New York: Chapman and Hall; 1993.

[6] Glaser R, Dimitrakakis C. Reduced incidence of breast cancer in women adherent to testosterone or testosterone-anastrozole hormone therapy: updated interim analysis. Maturitas. 2015;81(1):189.

[7] Feller WF. An Introduction to Probability Theory and its Applications. vol. 1. Wiley; 1950.

List of Tables

1 Beast cancer incidence rates from SEER 7
2 Whole sample summary statistics 8
3 Summary of bootstrap results 9
4 Confidence intervals and hypothesis tests 10
5 Estimated confidence intervals for Dayton and expected SEER cancer numbers with results of testing whether they are same vs that the Dayton numbers were less. 11

List of Figures

1 Bootstrap density of Dayton and expected SEER BC incidence rates. 12

Table 1: Breast cancer incidence rates per 100,000 py as reported by SEER for two time periods, 2007 – 2011 and 2011 – 2015. Standard deviations (SD) were reported for 2011 – 2015 but not for 2007-2011. SD's for 2007 – 2011 were estimated assuming that the coefficient of variation for the 2007-2011 values were the same as 2011-2015.

	Age-group	2007-2011	$\widehat{SD}$	2011-2015	SD	CV
1	15-19	0.16	0.00	–	0.00	–
2	20-24	1.45	0.14	1.60	0.15	0.0949
3	25-29	8.27	0.33	9.00	0.36	0.0397
4	30-34	26.24	0.61	27.30	0.63	0.0232
5	35-39	59.77	0.96	61.30	0.99	0.0161
6	40-44	120.48	1.29	124.60	1.33	0.0107
7	45-49	187.65	1.53	191.80	1.56	0.0082
8	50-54	230.63	1.61	230.00	1.60	0.0070
9	55-59	286.24	1.86	266.40	1.73	0.0065
10	60-64	353.33	2.12	347.10	2.09	0.0060
11	65-69	409.91	2.40	440.50	2.58	0.0059
12	70-74	425.54	2.81	476.60	3.15	0.0066
13	75-79	449.24	3.42	474.70	3.61	0.0076
14	80-84	430.48	3.78	432.50	3.79	0.0088
15	85+	365.05	3.17	348.70	3.03	0.0087

Table 2: Summary statistics, whole sample. For time-frames from 90 - 548 d, the number of observed cancers, the total number of person years, the Dayton incidence rate, the expected incidence rate if the SEER rates applied to the Dayton study, the SD of the expected incidence, the difference and the standard deviation between the Dayton and expected SEER rates, the ratio of the Dayton to the SEER incidence rate, and the expected number of cancers if the SEER rates were applicable.

Time Frame (days)	Number cancers	Person years	Dayton incidence	Dayton SD	SEER incidence	SD_1 SEER	SD_2 SEER	D	D SD	Ratio	SD Ratio	Seer cancers
90	6	6297.3	95.3	38.9	270.8	1.83	0.27	-175.48	38.94	0.35	0.14	17.05
120	7	6373.6	109.8	41.5	270.7	1.83	0.22	-160.88	41.55	0.41	0.15	17.25
150	9	6448.4	139.6	46.5	270.6	1.83	0.16	-131.07	46.56	0.52	0.17	17.45
180	10	6522.0	153.3	48.5	270.6	1.83	0.10	-117.26	48.52	0.57	0.18	17.65
210	11	6594.6	166.8	50.3	270.5	1.83	0.05	-103.73	50.33	0.62	0.19	17.84
240	11	6666.6	165.0	49.8	270.5	1.83	0.07	-105.48	49.78	0.61	0.18	18.03
365	12	6960.5	172.4	49.8	270.3	1.83	0.14	-97.95	49.80	0.64	0.18	18.82
548	12	7381.2	162.6	46.9	270.4	1.83	0.12	-107.79	46.97	0.60	0.17	19.96

Table 3: Bootstrap estimates summary statistics based on 10 000 bootstrap replicates: the mean number of cancers and person years, the Dayton and expected SEER incidence rates, their standard deviations, the mean difference between the Dayton incidence and SEER incidence rates (D) and its standard deviation, the ratio of the Dayton incidence rate to the SEER and its SD for various time frames. 90 - 540 d. Time frame is number of days post last pellet insertion.

Time frame (days)	Mean cancers	Mean person years	Dayton incidence	Dayton SD	Seer incidence	Seer SD	Mean difference	SD difference	Mean Ratio	SD Ratio
90	6.02	6296.8	95.6	38.57	270.8	3.1	-175.1	38.82	0.35	0.14
120	7.02	6374.3	110.3	41.72	270.7	3.1	-160.5	41.93	0.41	0.15
150	9.00	6448.2	139.6	47.10	270.6	3.1	-131.1	47.34	0.52	0.17
180	10.02	6521.3	153.7	48.78	270.6	3.0	-116.9	49.03	0.57	0.18
210	11.03	6594.8	167.3	50.11	270.5	3.0	-103.3	50.30	0.62	0.19
240	11.02	6665.6	165.4	49.85	270.5	3.0	-105.1	50.05	0.61	0.18
365	12.01	6960.2	172.7	49.45	270.3	2.9	-97.7	49.58	0.64	0.18
548	12.04	7382.4	163.2	47.06	270.4	2.8	-107.2	47.25	0.60	0.17

Table 4: Estimated confidence intervals for Dayton and expected SEER incidence rates and significance tests of difference.

Time frame	Dayton incidence	Dayton lci	Dayton uci	SEER incidence	SEER lci	SEER uci	P
Whole sample results							
90	95.28	34.97	207.38	270.76	267.17	274.35	< 0.0001
120	109.83	44.16	226.29	270.70	267.12	274.29	< 0.0001
150	139.57	63.82	264.95	270.64	267.06	274.23	0.001
180	153.33	73.53	281.98	270.59	267.00	274.18	0.004
240	165.00	82.37	295.23	270.48	266.90	274.07	0.008
365	172.40	89.08	301.15	270.35	266.76	273.94	0.012
Bootstrap results							
90	95.63	31.24	177.44	270.78	264.65	276.82	< 0.0001
120	110.26	31.82	200.18	270.73	264.63	276.80	< 0.0001
150	139.57	60.31	238.90	270.63	264.55	276.73	< 0.0001
180	153.75	62.59	257.01	270.62	264.75	276.46	< 0.0001
240	165.33	74.68	271.09	270.46	264.46	276.27	< 0.0001
365	172.65	85.02	275.88	270.33	264.51	276.03	< 0.0001

Table 5: Estimated confidence intervals for Dayton and expected SEER cancer numbers with results of testing whether they are same vs that the Dayton numbers were less.

ER cancers

Time frame	Dayton mean cancers	Dayton lci	Dayton uci	SEER mean cancers	SEER lci	SEER uci	P
Whole sample results							
90	6.00	2.20	13.06	17.05	16.82	17.28	0.0001
120	7.00	2.81	14.42	17.25	17.02	17.48	0.0004
150	9.00	4.12	17.08	17.45	17.22	17.68	0.0053
180	10.00	4.80	18.39	17.65	17.41	17.88	0.0143
240	11.00	5.43	19.47	17.84	17.60	18.07	0.0322
365	12.00	5.94	20.08	18.02	17.78	18.26	0.0374
Bootstrap results							
90	6.02	2.00	11.00	17.05	16.22	17.87	< 0.0001
120	7.02	2.00	13.00	17.26	16.43	18.08	< 0.0001
150	9.00	4.00	15.00	17.45	16.64	18.28	< 0.0001
180	10.02	4.00	17.00	17.65	16.84	18.48	< 0.0001
240	11.02	5.00	18.00	18.03	17.21	18.86	< 0.0001
365	12.01	6.00	19.00	18.82	18.02	19.63	< 0.0001

Figure 1: Density plot of bootstrapped estimates of the Dayton breast cancer and expected SEER incidence rates for several time-frames of “active therapy”. The gray box encloses the complete range of expected SEER rates and the black arrow is the location of the median value.

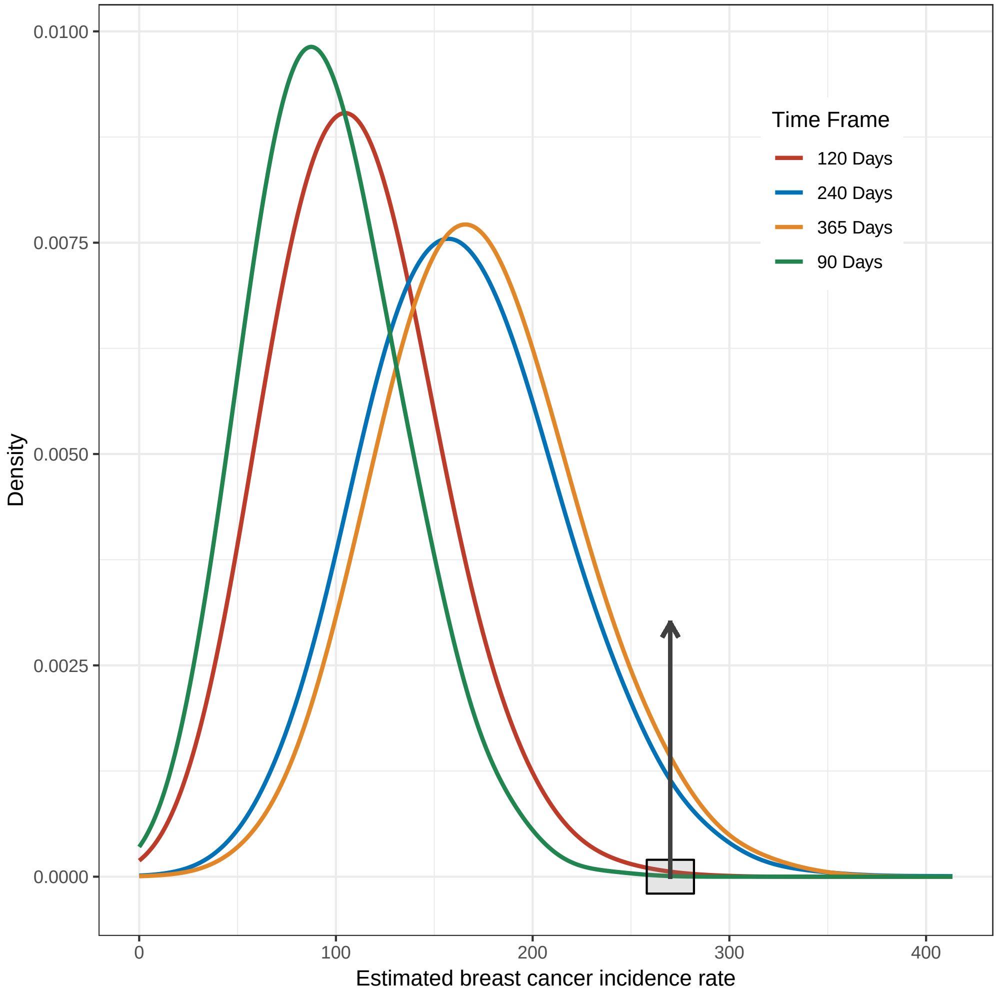

Supplement: Supplementary file 1 — Additional file 1. Statistical Methods and Results [file 12885_2019_6457_MOESM1_ESM.pdf]
